# Prevalence and impact of infant oral mutilation on dental occlusion and oral health-related quality of life among Kenyan adolescents from Maasai Mara

**DOI:** 10.1186/s12903-018-0631-2

**Published:** 2018-10-24

**Authors:** Arthur Kemoli, Hans Gjørup, Marie-Louise Milvang Nørregaard, Mark Lindholm, Tonnie Mulli, Anders Johansson, Dorte Haubek

**Affiliations:** 1 Department of Paediatric Dentistry, University of Nairobi, Nairobi, Kenya; 2 Center for Oral Health in Rare Diseases, Department of Maxillofacial Surgery, Aarhus University Hospital, Aarhus C, Denmark; 3 Section for Pediatric Dentistry, Department of Dentistry and Oral Health, Health, Aarhus University, Aarhus C, Denmark; 4 Division for Oral Microbiology, Odontology, Umeå University, Umeå, Sweden; 5 Department of Periodontology, University of Nairobi, Nairobi, Kenya; 6 Molecular Periodontology, Odontology, Umeå University, Umeå, Sweden

**Keywords:** Tooth bud, Germectomy, Avulsion, Ebinyo, Malocclusion, Life quality

## Abstract

**Background:**

Infant Oral Mutilation (IOM) includes germectomy and early extraction of primary and permanent incisors and canines, primarily in the lower jaw.

The aim of the present study was to examine the prevalence and impact of IOM, involving the removal of mandibular permanent incisors and/or canines, on dental occlusion and Oral Health-Related Quality of Life (OHRQoL) among Kenyan adolescents from Maasai Mara.

**Methods:**

In a cross-sectional study, 284 adolescents (14–18 yrs. of age) participated in an oral examination and an interview, using a structured questionnaire on age, gender, medical history, and IOM practice. For the analysis of the dental occlusion, participants with IOM, in terms of absence of two or more permanent teeth in the mandibular incisor and/or canine tooth segments (IOM group), were compared to participants who had all six incisors and canines present in the oral cavity (control group). OHRQoL was assessed using child perception questionnaire (CPQ11–14).

**Results:**

The majority of the participants (61%) had been exposed to IOM, among whom 164 (95%) had absence of two mandibular central incisors. More individuals in the IOM group had maxillary overjet exceeding 5 mm than in the control group (50.9% vs. 20%, *p* <  0.001). Nineteen (11%) subjects in the IOM group had mesial occlusion in contrast to none in the control group (*p* <  0.001). The mean and median total CPQ scores and the mean and median CPQ domain scores were low in both groups with no significant differences between the groups.

**Conclusions:**

Approximately two-thirds of the study population presented with IOM, with the majority of them missing two mandibular permanent central incisors. Although some participants with IOM had substantial maxillary overjet and mesial occlusion, only few of them showed substantial effect on their OHRQoL.

## Background

Infant oral mutilation (IOM) is a traditional practice performed in young children, mostly as germectomy of developing primary or permanent mandibular incisors or canines, or early extraction of these tooth types [1–5]. The rationale for IOM can be either therapeutic or ritual [6, 7]. Beyond the removed teeth, dental defects, dental deficiency (aplasia of succedaneous permanent teeth due to IOM on primary teeth), and eruptional disturbances may occur [1, 3, 8]. In addition to these adverse defects and disturbances, unwanted side-effects on dental occlusion may occur due to imbalance of the space in the dental arches as, e.g., development of deep bite by overeruption of the upper incisors without antagonists [9, 10].

IOM is still rampant in several countries in the East African region and has been associated with geographic, cultural, aesthetic, and ritual grounds [1, 5, 8, 11–18]. For example, previous studies in Kenya demonstrate that various types of IOM are still practiced by some tribes in the country [15, 19]. A study by Hassanali and coworkers in a Maasai population from the Kajiado area reported a very high prevalence of removal of primary canine tooth buds in the age group 6 months to 2 years as well as in the age group 3 to 7-years of age (87% and 72%, respectively) [15]. In addition, traditional extraction of mandibular permanent central incisors in Maasai children has been demonstrated [20]. IOM has also been shown to affect the dental arch width [20], the development and eruption of the succedaneous teeth [9], and the dental occlusion [21]. In Kenya, apart from the observations made by Hassanali and coworkers [20], no other studies on the assessment of the long-term effects of IOM on the dental occlusion of the affected children have been found.

Currently, human migration from one part of the world to another is a relatively frequent event [22]. Therefore, subjects with IOM may appear geographically widespread, and hence the phenomenon is of relevance to clinicians all over the world.

The aim of the present study was to examine the prevalence and impact of IOM, involving the removal of mandibular permanent incisors and/or canines, on dental occlusion and Oral Health-Related Quality of Life (OHRQoL) among Kenyan adolescents from Maasai Mara.

## Methods

### Study population

The study was conducted in January–February 2016 and took place in Mara North Conservancy in Narok County of Kenya. Mara North Conservancy was established in January 2009 through a partnership among eleven member camps and over 800 Maasai landowners with long-term commitments to the environment, wildlife, and local communities.

The study population consisted of adolescents aged 14 to 18 years. They were recruited from the four primary and one mixed secondary schools present in Mara North Conservancy. Out of the total number of teenagers in this age group (*n* = 340), 284 (83.5%) teenagers [mean age: 15.0; SD 1.1; range 14–18 years] were recruited into the study. These were teenagers whose parents/guardians provided a written informed consent for their participation in the study. The teenagers, not included in the study, were those who failed to provide the consent, were absent, or sick on the day of the examination. The age of the participants was determined from the records kept by the schools, except for three of the teenagers, whose age records were missing in the school register. The distribution of the participants according to gender was 153 (55.6%) males and 122 (44.4%) females (information on gender had unintentionally been omitted in the record sheet for nine teenagers). Information on social and economic status of the teenagers and their families was not available to the researchers. The few schools (*n* = 5) in Mara North Conservancy, Narok County, are boarding schools, as the possibilities for transportation within the region is scarce and challenging. Thus, most often parents live far away from the schools. All schools were considered to be at a similar standard and with similar physical and educational possibilities.

The study consisted of two parts, one being a face-to-face interview with the teenagers using structured questionnaires to collect data on age, gender, medical history, IOM practice, and OHRQoL, while the second part included an examination of the participants` teeth present in the oral cavity, including oral photographing of the dentition.

### Face-to face interview

Structured questionnaires were used to collect data on age, gender, medical history, IOM practice, and OHRQoL. In order to prevent copying of answers to the questionnaire amongst the participants from the same school class, a clear separation method was applied to prevent intermingling of the participants, until the interviews were finalized.

The OHRQoL part was assessed by the validated Child Perception Questionnaire (CPQ11–14), which is developed to measure the OHRQoL among teenagers [23, 24]. The CPQ includes 37 questions grouped into four domain subscales: oral symptoms, functional limitations, emotional well-being, and social well-being. The response format for all questions is a Likert-like scale. The response options and scores are: “never” (score 0), “once or twice” (score 1), “sometimes” (score 2), “often” (score 3) and “every day or almost every day” (score 4). The range of the additive total CPQ score is 0–148. The ranges of domain subscale scores are 0–24 (oral symptoms), 0–36 (functional limitations and emotional well-being), and 0–52 (social well-being). In addition, the CPQ includes two global questions: Q1) “How would you describe the healthiness of your teeth, mouth, lips or jaws?” (very good, good, okay, or bad) and Q2) “How much does the condition of your teeth, mouth, lips or jaws influence your life?” (not at all, very little, some, a lot, or very much).

The questionnaire for the collection of data on age, gender, medical history, and IOM practice was initially piloted and tested by the two Kenyan authors (AK and TM) concerning the understandability and relevance in a Kenyan context before being used. Further, the Kenyan authors were also the dentists who had the contact with the teenagers when they were interviewed, meaning that the teenagers had the possibility to ask probing questions in English or local languages. The original CPQ questionnaire is written in English [23, 24], and the spoken language in Kenya is English. The English CPQ questionnaire has been validated in other English-speaking communities [23, 24], but it has not been validated specifically in the Kenyan population. As a supplement, the CPQ questionnaire was also translated to the local tribe language of the Maasai population, in case a need arose of having the English version of some or all the questions in the local language for clarification. In addition, the participants did not fill out the questionnaire themselves, but the procedure was carried out by the interviewer and any assistance, if needed, was available from the Kenyan co-authors of the present paper. In practice, there was, however, no need for the translated questionnaire as only probing questions were asked by some participants and subsequently explained by the interviewers. The two interviewers were Kenyan dental researchers from University of Nairobi, Kenya, and they were trained in using the questionnaires, and in addition, they calibrated the interview procedure under field conditions after the finalization of the initial two interviews.

### Oral examination

The oral examination was done under field conditions at the respective schools of the teenagers. This means that oral examinations were not performed in a dental office, but in a standard class room with natural lighting. No sophisticated dental equipment was available. The child was made to lie on the top of a table, facing a natural light source. As supplementary light source, a headlamp was used to augment the natural light during the examination of the oral cavity. With clean disposable mouth mirrors and tweezers, an oral examination was carried out to establish the status of the dentition and the dental occlusion. A record on the number of teeth present in the mandibular incisor and canine segments and signs of dental disruption was made on individual forms. Teeth were recorded as present when either partly or fully erupted. A tooth was recorded as having a dental disruption, if the tooth had an abnormal and irregular morphology with unusual hypoplastic defects consistent with previous germectomy in the affected area of the dental arch. Thus, dental disruption was defined as an extrinsic hypoplastic defect or interference with the normal developmental process of the tooth. Dental fluorosis was seen in the study population, but was not an aim to study in the present study. An IOM case was defined as an individual who was missing two or more permanent teeth in the mandibular incisor and/or canine tooth segments, as a result of IOM (also confirmed during interview). Intraoral photographs were taken as a part of the record, with the teeth in occlusion from right, left, and frontal perspective.

The dental occlusion was assessed according to definitions by Bjoerk, Krebs and Solow [25] and included measurement of the horizontal overjet (HO) and the vertical overbite (VO) with a caliper, classification of HO into mandibular overjet (HO ≤ 0 mm), neutral overjet (0 mm < HO ≤ 5 mm), maxillary overjet (5 mm < HO < 9 mm), or extreme maxillary overjet (HO ≥ 9 mm), and classification of VO into neutral overbite (0 mm ≤ VO ≤ 4 mm), deep bite (overbite ≥5 mm), or frontal open bite (VO <  0 mm). Furthermore, the molar occlusion on each side of the participants was assessed and classified as neutral (the mesiobuccal cusp of the maxillary permanent first molar occludes into the mesiofacial sulcus of the mandibular permanent first molar), distal (mandibular first molar deviates distally to neutral occlusion ½ cusp or more), or mesial (mandibular first molar deviates mesially to neutral occlusion ½ cusp or more). For each side, deviations from normal transverse occlusion was classified as cross bite (the buccal cusp of at least one maxillary canine, premolar, or molar occludes lingual to the buccal cusp of the mandibular teeth) or scissor bite (the lingual cusp of at least one maxillary canine, premolar or molar occludes buccal to the buccal cusps of the mandibular teeth).

Prior to the initiation of the study, training of the researchers, to standardize the methods to be applied, was carried out by studying pictures available in the published literature as well as clinical photos taken of the participants on the first day of the study period. Due to the limited working time at the research site, recall of patients for traditional intra-reliability evaluation was not an option. Only two dentists examined the children (HG, MLMN), while two other dentists (ML, DH) did the recording of the results and the oral photographing. Concerning the inter-rater reliability, the two clinical examiners did an examination twice of 12 participants randomly chosen among the 284 participants. The examinations done twice were executed with four students at the initiation of the study and with two participants another four times during the remaining part of the study. A maximum of (12 × 32 teeth) 384 teeth were included in the double examinations among which a total of 327 (85.1%) were actually found to be present in the oral cavity. Concerning the recording of the teeth present in the oral cavity and the teeth with dental disruption, the percentage agreement between the two examiners were 100%. The missing teeth recorded during the 12 examinations were 35 third molars, 4 s permanent molars, 15 mandibular permanent central incisors, two mandibular permanent canines, and one maxillary permanent canine.

All the children at the participating schools received free education on oral hygiene with a toothbrush and toothpaste provided to them for continued use in school/at home. The participants, who required emergency dental treatment, were referred to the nearest dental clinic or the Dental Hospital of the University of Nairobi.

### Data analysis

The data collected were cleaned, coded, and entered into the computer, and analyzed with the use of SPSS 24 (Statistical Package for the Social Sciences, SPSS Inc., Chicago, IL) and STATA 14.0 (StataCorp LLC, Texas, USA). The number of maxillary teeth was compared to the number of mandibular teeth. The total number of missing maxillary incisors and canines was compared to the total number of missing mandibular incisors and canines. For studying the potential consequences of missing teeth due to IOM in the anterior segment of the mandible in relation to the dental occlusion, the IOM group was defined as participants with two or more missing mandibular incisors and/or canines. The group of participants, in whom all mandibular canines and incisors were present, was defined as the control group. Sixteen participants with the absence of only one mandibular permanent incisor or canine were excluded from the comparison between groups due to one missing tooth being below the defined cut-off level.

Overall CPQ11–14 score and domain scores for each participant were calculated by summing the response codes for the questions. If one or more of the questions in a domain were unanswered, the respective domain score as well as the overall CPQ11–14 score was recorded as missing for that participant. The mean additive score of each domain as well as the mean overall CPQ11–14 score were calculated and indicate the severity of impact on OHRQoL in the respective domains [26]. For the CPQ11–14 scale as a whole and for each of the four domains, the number of answers, being reported as “often” or “every-day/almost every day”, were counted. The mean of these figures indicate the extent of severe impact on OHRQoL in the respective domains. The percentage of individuals answering “often” or “every-day/almost every day” was calculated and indicate the prevalence of severe impact on OHRQoL in the respective domains [26]. In addition, the median additive scores in the respective domains as well as the median overall CPQ11–14 score were calculated due to the scores not being normally distributed.

Deviations on the dental occlusion and in the answers on IOM and CPQ were assessed according to the defined grouping of participants with or without IOM.

Statistical tests in terms of t-test, Wilcoxon rank sum test (Mann-Whitney), Fischer’s exact test, and Chi-square were carried out as appropriate.

## Results

### Number of teeth present in the oral cavity

Among 283 out of 284 teenagers entered into the study, the overall mean number of permanent teeth present in the oral cavity was 27.9 [SD: 2.0; range: 22–32; 95% CI: 27.7–28.1]. The calculation was based on 283 adolescents only, as one individual, who had only 11 permanent teeth and multiple primary teeth present (most likely due to delayed eruption), was excluded from the calculation of the mean number of the permanent teeth present, but not from other calculations in the study. The number of maxillary teeth [mean 14.5; SD 1.1; 95% CI: 14.4–14.6] exceeds the number of mandibular teeth [mean 13.4; SD 1.3; 95% CI: 13.2–13.5] (*p <* 0.001). The total number of missing mandibular incisors and canines [mean 1.4; SD 1.1; 95% CI: 1.2–1.5] exceeds the total number of missing maxillary incisors and canines [mean 0.1; SD 0.4; 95% CI: 0.1–0.2] (*p <* 0.001).

**Fig. 1:**
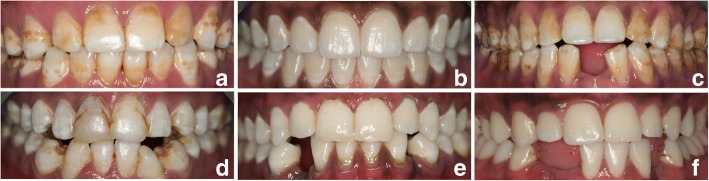
Kenyan teenagers without IOM (**a** and **b**) and with IOM (**c**, **d**, **e** and **f**). Examples given in **c** and **d** illustrate the traditional type of IOM (two mandibular incisors missing) among adolescents living in Maasai Mara, and the vast majority of the study population (61%) presented with this type of IOM. Space between teeth is seen between mandibular lateral incisors in case C, whereas in case D the space has been closed after removal of mandibular incisors. Cases E and F show uni- and/or bilateral missing permanent canines and/or incisors. Dental fluorosis (variation in severity) is seen on the pictures

The distribution of clinically visible teeth as well as the absence of teeth in the mandible according to tooth type is provided in Table 1. A total of 173 out of 284 (61%) teenagers belonged to the IOM group, with bilateral absence of the mandibular central incisors being the dominant finding in relation to the IOM practice (164 out of 173 subjects in the IOM group (94.8%) and 164 out of 284 in the total group (57.7%)) (Fig. 1c and d). Concerning permanent molars, 107, 277 and 276 individuals, respectively, had third molars, second molar and first molars bilaterally present. Third, second, and first permanent molars were absent bilaterally in, 154, three, and two individuals, respectively. Twenty-one, four, and six individuals, respectively, had this status unilaterally.

**Table 1:** Presence of permanent and primary mandibular teeth and occurrence of dental disruption according to tooth type (*n* = 284)

	DP^a^ present	DP absent		dd^b^ present	DP with disruption	
Mandibular tooth type	bilateral (n)	bilateral (n)	unilateral (n)	(n)	bilateral (n)	unilateral (n)
Second premolar	280	0	1	3	0	0
First premolar	282	0	1	1	1	0
Canine	267	5	10	2	0	6
Lateral incisor	263	5	14	2	0	3
Central incisor	108	164	12	0	0	0

^a^DP means permanent teeth

^b^dd means primary teeth

### Disruption of teeth

The distribution of mandibular premolars, canines and incisors with disruption of the tooth crown is also shown in Table 1. Eight individuals (8/284 (2.8%)) had a total of 11 mandibular premolars, canines, and/or incisors with disruption of the tooth crown. Specifically, one individual had dental disruption of three (tooth no. 34, 44, and 33), one individual had disruption of two (tooth no. 43 and 42), and six individuals had disruption of one tooth crown (three individuals: tooth no. 43; two individuals: tooth no. 32; one individual: tooth no. 33). Thus, in summary a total of 8 individuals had disruption of one or more teeth in the incisor, canine and premolar tooth segments of the mandible.

### Dental occlusion

The characteristics of the dental occlusion according to IOM or control group are shown in Table 2. More individuals in the IOM group had maxillary overjet exceeding 5 mm than in the control group (86 (50.9%) vs. 19 (20%), *p* < 0.001). Nineteen (11%) subjects in the IOM group had mesial occlusion in contrast to none in the control group (*p* < 0.001), whereas no significant difference was seen according to findings of distal occlusion, cross bite, and scissor bite. There was no significant difference found in relation to the categories of VO (neutral overbite, deep bite, and frontal open bite) when comparing the IOM group and the control group.

**Table 2:** Characteristics of dental occlusion in the infant oral mutilation (IOM) group compared to the control group

	IOM group (*n* = 173)^a^	Control group (*n* = 95)	*p*
	Number (%)	Number (%)	
Mandibular overjet (HO ≤ 0 mm)	1 (0.6)	0	< 0.001
Neutral overjet (0 < HO ≤ 5 mm)	83 (49.1)	76 (80.0)	
Maxillary overjet (5 < HO < 9 mm)	52 (30.8)	17(17.9)	
Extreme maxillary overjet (HO ≥ 9 mm)	34 (20.1)	2 (2.1)	
Neutral overbite (0 ≤ VO ≤ 4)	139 (83.7)	85 (89.5)	0.226
Deep bite (VO ≥ 5 mm)	17 (10.2)	4 (4.2)	
Frontal open bite (VO < 0)	10 (6.0)	6 (6.3)	
Molar occlusion			
Mesial (one or both sides)	19 (11.0)	0	< 0.001
Distal (one or both sides)	4 (2.3)	4 (4.2)	0.382
Cross bite (one or both sides)	14 (8.1)	12 (12.6)	0.230
Scissor bite (one or both sides)	5 (2.9)	3 (3.2)	0.902
	Mean (SD) [95% CI]	Mean (SD) [95% CI]	*p*
Horizontal overjet (mm)	5.9 (2.8) [5.5–6.4]	4.1 (SD 1.9) [3.7–4.5]	< 0.001
Vertical overbite (mm)	2.3 (2.4) [1.1–2.6]	2.0 (SD 1.8) [1.6–2.3]	0.298

Comparison by Chi^2^-test (HO categories, VO categories, and molar occlusion categories) or t-test (mean HO and mean VO)

Figures in parentheses are percentages of patients with the deviation in the group

Figures in brackets [] are 95% confidence interval (CI)

^a^Missing data on HO of four patients and on VO of seven patients

IOM group: Teenagers missing two to four mandibular incisors and/or canines

Control group: Teenagers with all mandibular incisors and canines present

The mean HO was significantly higher in the IOM group compared to the control group (*p* < 0.001), whereas no significant difference in mean VO was found (*p* = 0.298).

### Answers to questions on IOM practice

The answers on the subjective aspects of IOM by the 173 (61.1%) teenagers, who had entered the IOM group, are summarized in Table 3. The information on the age at the time of tooth extraction was missing in most cases (*n* = 137). Thus, the possibility that IOM had been carried out at a very early age, exists. The mean age reported as the time point of the extraction for the group (*n* = 36), who remembered the age/occasion, was 7.7 yrs. [SD: 7.7 yrs.; range 3–12 yrs].

**Table 3:** The answers on aspects related to tooth removal given by 173 adolescents with infant oral mutilation (IOM)

Questions asked	Answers given to questions asked (number (%))				
“Who removed teeth?”	dentist	healer	other person	do not know	not recorded
	4 (2.3)	21 (12.3)	90 (52.0)	57 (33.0)	1 (0.6)
“Which tool was used to remove teeth?”	nail/needle	knife	other	do not know	not recorded
	0 (0)	93 (53.8)	23 (13.3)	55 (31.8)	2 (1.2)
“Who brought you for tooth removal?”	parents	friends	other	do not know	not recorded
	104 (60.1)	0 (0)	6 (3.5)	60 (34.7)	3 (1.7)
“How do you like^a^ your teeth?”	happy	do not like (miss)^b^	do not like (other)^c^	do not know	not recorded
	139 (80.4)	27 (15.6)	7 (4.1)	0 (0)	0 (0)
“Why was tooth removal carried out?”	ritual	esthetic	sick	do not know	not recorded
	151 (87.3)	1 (0.6)	1 (0.6)	19 (11.0)	1 (0.6)
“Is pain control used during tooth removal?”	no	yes		do not know	not recorded
	103 (59.5)	5 (2.9)		62 (35.8)	3 (1.7)
“Is tooth removal a tribe tradition?”	no	yes		do not know	not recorded
	170 (98.3)	0 (0)		2 (1.2)	1 (0.6)
“Is tooth removal a family practice?”	no	yes		do not know	not recorded
	146 (84.4)	24 (14.0)		3 (1.7)	0 (0)
“Is tooth removal seen also in siblings?”	no	yes		do not know	not recorded
	21 (12.3)	151 (87.3)		1 (0.6)	0 (0)

Figures given are numbers of adolescents with the specified answer, and the figures in parentheses are percentages of the total group (*n* = 173)

IOM: Absence of a minimum of two mandibular incisors and/or canines according to the cut-off level

^a^The word “like” means “wish to have”/“to take pleasure with”

^b^“I do not like that I have missing teeth in the front”

^c^“I do not like the esthetics of my teeth for other reasons than having missing teeth”

The two questions, dealing with the type of person who carried out the tooth removal and how the tooth removal was performed, were in about one third part of the participants answered by “don’t know” (31.8% and 35.8%, respectively). Pain control was not used in 60% of the cases. In 87% of the cases, tooth removal was practiced also in siblings. The majority of the adolescents considered tooth removal to be executed for ritual reasons (84%), but in most cases (98%) the participants did not consider tooth removal as a tradition in neither the tribe nor the family. Overall, the majority of the participants (80%) felt happy about the status of their teeth (Table 3).

### Answers to CPQ

The numbers of participants completing the specific measures (domains) are given in Table 4. Some answers were missing due to some teenagers refusing to answer the question. In the IOM group, the number of individuals with missing domain scores were respectively two (oral symptoms), five (functional limitations), three (emotional well-being), and one (social well-being). In the control group, the number of individuals with missing domain scores were respectively one (oral symptoms) and two (functional limitations).

**Table 4:** The overall CPQ11–14 score and the four domain scores in 173 adolescents infant oral mutilation (IOM group) compared to 95 adolescents with all mandibular incisors and canines present in the oral cavity (control group)

		CPQ total				Oral symptoms				Functional limitations				Emotional well-being				Social well-being			
	n^a^	Median^b^ (P^10^-P^90^)	Mean^c^ (SD)	Preva-lence (%)^d^	Extent^e^	Median^b^ (P^10^-P^90^)	Mean^c^ (SD)	Preva-lence (%)^d^	Extent^e^	Median^b^ (P^10^-P^90^)	Mean^c^ (SD)	Preva-lence (%)^d^	Extent^e^	Median^b^ (P^10^-P^90^)	Mean^c^ (SD)	Preva- lence (%)^d^	Extent^e^	Median^b^ (P^10^-P^90^)	Mean^c^ (SD)	Preva-lence (%)^d^	Extent^e^
IOM group	173	4 (0–18)	6.0 (8.0)	5.5	0.12	2 (0–5)	2.4 (2.3)	3.5	0.04	1 (0–6)	2.1 (3.4)	5.4	0.07	0 (0–3.5)	0.9 (2.9)	1.2	0.02	0 (0–2)	0.7 (2.4)	0.6	0.01
Boys	96	4 (0–20)	7.0 8.9	7.6	0.18	2 (0–6)	2.8 (2.4)	4.3	0.05	1 (0–8)	2.3 (3.5)	6.5	0.09	0 (0–4)	1.0 (3.1)	2.1	0.03	0 (0–2)	0.8 (2.5)	1.0	0.01
Girls	72	4.5 (0–16)	7.6 (8.9)	2.9	0.03	2 (0–6)	2.8 (2.4)	2.8	0.03	1 (0–9)	2.8 (3.7)	4.3	0.06	0 (0–5)	1.2 (2.9)	0	0	0 (0–6)	1.2 (2.8)	0	0
Control group	95	4 (0–14)	5.9 (7.2)	4.4	0.05	2 (0–6)	2.5 (2.2)	3.2	0.04	0 (0–7)	2.1 (3.0)	1.1	0.01	0 (0–2)	0.9 (2.2)	0	0	0 (0–2)	0.7 (2.0)	0	0
Boys	48	4 (0.11)	4.6 (5.0)	2.1	0.02	2 (0–6)	2.4 (2.0)	2.1	0.02	0 (0–6)	1.5 (2.3)	0	0	0 (0–2)	0.6 (1.5)	0	0	0 (0–0)	0.2 (0.7)	0	0
Girls	44	2 (0–13	4.6 (6.1)	7.1	0.10	2 (0–4)	1.9 (2.0)	4.7	0.07	0 (0–5)	1.9 (3.3)	2.3	0.02	0 (0–2)	0.7 (2.5)	0	0	0 (0–0)	0.5 (2.0)	0	0

^a^The number of individuals in the respective groups. Missing data on gender of five IOM individuals and three controls

^b^Median additive score, 10- and 90-percentiles in parenthesis

^c^Mean additive score, standard deviation (SD) in parenthesis (severity of impact)

^d^Prevalence is the percentage of individuals with one or more items scored “often” or “every day/almost every day” in the specified domains

^e^Extent is the mean number of items scored “often” or “every day/almost every day” in the specified domain

IOM: Absence of a minimum of two mandibular incisors and/or canines according to the cut-off level

The healthiness of teeth and mouth (Q1) was characterized as “very good” or “good” in contrast to “okay” or “bad” by 148 (86%) individuals in the IOM group and by 83 (87%) individuals in the control group (*p* = 0.853). How much the condition of teeth and mouth influenced their lifes (Q2) was answered by “not at all” or “very little” in contrast to “some”, “a lot”, or “very much” by 156 (91%) individuals in the IOM group and by 85 (89%) in the control group (*p* = 0.665). The mean and median total CPQ scores and the mean and median domain scores were low in both groups, and no significant differences between groups were found (*p* ≥ 0.191) (Table 4).

## Discussion

The present research project took place in Maasai Mara North Conservancy, a rural Kenyan area that forms part of the Maasai Mara, where the Maasai Mara National Park is situated. The area was chosen as the research site, because it was part of a larger interdisciplinary research project under the auspices of The Maasai Mara Science and Development Initiative (http://maasaimarascience.org/). The indigenous Maasai population living in the area still maintains their traditional life, although human wildlife interaction can be challenging in addition to the interaction with the tourists visiting the national park. It is plausible to expect some changes in the traditions of the Maasai population due to such interactions.

Absence of two mandibular central incisors as a sign of IOM was found in the majority of the teenagers living in Maasai Mara, which was an indication of IOM, in terms of removal of tooth buds or early extraction of mandibular incisors, still being a very common practice in the Maasai Mara area. Other causes than IOM to explain the absence of mandibular incisors could not be fully excluded. The absence of some of the mandibular incisors may theoretically be because of dental anomaly, e.g., agenesis of lower incisor(s), deviation of the dental eruption, e.g., retention or impaction of incisors, or avulsion because of traumatic injury. In other populations, agenesis of mandibular incisors is, however, a very rare finding (95% CI: 0.25–0.35%) in comparison to agenesis of mandibular second premolars (95% CI: 2.91–3.22%), maxillary second premolars (95% CI: 1.39–1.61%), and lateral maxillary incisors (95% CI: 1.55–1.78) [27]. Also avulsion of mandibular incisors is rare [28, 29]. Thus, the absence of mandibular permanent incisors found in the present study is most likely explained by IOM. We had, however, only minimal or no information on the dental history of the participants, and radiographic equipment was not available at the research site in Maasai Mara.

Other types of IOM than absence of two mandibular central incisors were also found, for example, a combination of missing lateral incisors and canines (Fig. 1). These types were, however, much less common. According to the present study, the IOM practice impacts on OHRQoL and the dental occlusion to a minor extent only, and according to the questions and aspects assessed in the study, the teenagers were in general satisfied with their dental status.

In the present study, the prevalence of IOM was found to be high (61%). This finding was much higher than the findings in a Sudanese study, reporting 22.4% of children (aged 4 to 8 years) having IOM [30], and in an Ethiopian study, reporting 15% of 2 to 18-year old children having IOM in terms of primary canines extraction and 7% of their permanent canines being affected by the traditional IOM practice [14]. In terms of the missing teeth due to IOM, the present study found the mandibular central incisors to be the most frequently affected tooth type. This result is different from the two above mentioned studies [14, 30], which involved mostly the canines. In contrast, the findings of the present study support previous reports from Maasai Mara, which also describes the absence of mandibular incisors as a dominant and characteristic IOM trait in the Maasai population [7, 20].

In the present Kenyan study, signs of dental disruption during the development of the tooth crowns was seen in few teeth (incisors, canines and/or premolars), and only a minor proportion of the study population (2.8%) showed this deviation of the tooth formation in the mandible. In the previously mentioned Sudanese study on IOM (termed “haifat”), the mandibular permanent canines were found to be the most affected tooth type, primarily with enamel defects on the labial surfaces [30]. In the Sudanese study, 28.4% of the children with IOM had enamel defects compared to only 8.4% among the controls. In a Tanzanian study, the prevalence of missing and/or disrupted permanent teeth was 8% [12]. All these studies affirm the fact that there is a high risk of damage to tooth germs of permanent teeth while removing other tooth buds or doing early extractions. Dental disruption can be the result of the use of improper instruments to undertake the IOM procedure [3]. Besides the reasons given above, the lack of aseptic procedures could result in local or general infection during the critical period of tooth development and mineralization [19]. This could also result in enamel defects of the tooth crowns. Moreover, a likely explanation to the dental disruption seen on premolars is that ‘neighboring’ tooth bud(s) to the tooth bud/tooth that was intended to having IOM done, were “hidden” and thereby also damaged, most likely unintentionally.

Dental fluorosis was seen prominently on all teeth of the vast majority of the children participating in the study. Dental fluorosis is endemic in Kenya [31, 32], including the area of Mara North Conservancy. In cases with dental fluorosis, an atypical discoloration of the enamel (from white to brown), is seen. Severe dental fluorosis can, in addition, lead to disintegration of the tooth enamel [33]. However, dental disruption is a quantitative enamel defect, whereas dental fluorosis is a qualitative defect of enamel, eventually complicated by post-eruptive enamel breakdown due to less robust quality of enamel [32]. This circumstance also may need to be taken into consideration while diagnosing tooth anomalies in the population living in Maasai Mara, Kenya. The finding of IOM and enamel defects are, however, not so surprising in the Maasai Mara area, as it is relatively remote and lacks access to the requisite health facilities and oral health education [19].

IOM undertaken as germectomy or early extraction has been found not only to lead to dental disruption of succedaneous or adjacent teeth, but also to affect dental arch width. This has been reported in a study where the oral mutilation involved the extraction of mandibular central incisors [20]. In the present study, the dominant occlusal deviation in the group of participants, who had undergone mandibular incisor removal, was the increased maxillary overjet when compared to the controls without any tooth removal. The difference was statistically highly significant (Table 2), but the overall consequences on the dental occlusion appeared to be at a low to moderate level. However, the presence of mesial molar occlusion is relatively prevalent in the IOM group in contrast to the low prevalence of distal molar occlusion in both IOM group and control group. In Caucasian populations, distal molar occlusion is much more prevalent than mesial occlusion, e.g., in a previous Scandinavian study, which describes mesial molar occlusion in 3–4% and distal molar occlusion in 23–26% of an adolescent population [33, 34]. The prevalent mesial molar occlusion in the IOM group is most likely explained by mesial migration of mandibular teeth after the removal of teeth in the anterior segment of the lower dental arch. Normally, mesial molar occlusion is associated with mandibular overjet, which was present in only one individual of our study population. In general, an increased overjet is associated with distal molar occlusion [34], which was a rare finding in our study group. Thereby, the increased overjet does not seem to be associated with a total retrusion of the mandible or the lower dental arch, but may be explained by a constriction of the anterior segment of the lower dental arch due to removal of incisors in combination with a proclination of the maxillary incisors, eventually because of a forward positioning of the tongue. However, it might be speculated that IOM in terms of incisor removal impacts less on dental occlusion than the absence of canines. In case of missing canines, the occlusal consequences are most likely more extensive. This topic needs to be explored further in a population, where removal of canines is the dominant type of IOM.

In the present study, the exact time when IOM was carried out, was not known, and only a minor proportion of the participants could remember who had performed the IOM (14.3%). However, more than half of the subjects did remember the knife as the tool likely to having been used (Table 3). These findings could be due to the fact that in the majority of the children, the extraction was done early in life. Therefore, they may not be able to recall the incident. Furthermore, the present study showed that the majority (59.5%) of the participants remembered that no form of anesthetics or pain killer tablets was used to obtain pain control. The lack of pain relief may bring children in a condition where they are not able to participate safely in IOM procedures, which could lead to further trauma of other adjacent oral structures. Furthermore, the reason for the dental mutilation carried out might not have been clear to the growing children due to their immaturity. But the majority of teenagers (87.3%) thought that the incident might have been carried out because of tradition or as a ritual. Thus, it was not surprising that the majority of the participants did indicate that their siblings also had experienced tooth removal.

In terms of the effects of IOM on the teenagers daily functioning, most of the teenagers (80.4%) were happy with their dentition irrespective of signs of IOM. Thus, IOM does not seem to have a considerable effect on the OHRQoL. As mentioned in the method section, in order to prevent copying of answers to the questionnaire amongst the participants from the same school class, a clear separation method was applied to prevent intermingling of the participants, until the interviews were finalized. This organization is likely to be a strength of the data collection procedure increasing the validity of the collected data.

The participants came from Mara North Conservancy and were part of the Maasai population with a semi-nomadic lifestyle. The present study sample represents the population living in Maasai Mara only and cannot be extrapolated to Kenya in general. Experienced dental professionals within their dental field collected the data. The clinical examinations were done under field conditions (in class rooms in schools) where lighting was of various quality. This might have affected the results to some extent. However, clinical photos taken were useful as diagnostic supplement to the clinical data collected during the clinical examinations. The lack of radiographic facilities in the area excluded the possibility of diagnosing dental agenesis, impaction of teeth, un-erupted teeth, and other intraosseous structures or pathologies. Nevertheless, except for one subject, all participants had a fully or nearly fully matured permanent dentition minimizing the diagnostic uncertainty due to lack of radiographic equipment. But theoretically, the absence of teeth in the anterior tooth segment of the mandibular arch might be due to other reasons than removal or extraction of incisors. However, previous studies from Maasai Mara have reported on extraction of mandibular incisors as a common tradition [20]. Thus, the vast majority of the absent incisors is likely to be due to germectomy or early extractions.

Oral health education to the community to increase the understanding of the possible long-term effects of IOM practice is needed. This could be done with help from the community health workers and leaders. In addition, there is a need for further studies on appropriate strategies that could be used to “demystifying” the practice and for the development of relevant oral health education programs to address this issue in the tribes that still practice IOM. Future research may include studies on the dental status in young children and qualitative studies focusing attitude to and experiences of IOM in groups of mothers/parents, and elderly people of the Kenyan population. Furthermore, long-term consequences on dental occlusion in populations, where removal of primary and permanent canines are prevalent, need to be explored further, as that type of IOM may impact differently on the dental occlusion than IOM with removal of mandibular incisors.

## Conclusions

IOM is still very common in the Maasai Mara region with the extraction of the mandibular central incisors being the most dominant type of IOM. The consequence of the removal of mandibular central incisors is apparently minimal in relation to the dental occlusion and OHRQoL, although some, of course, are more heavily affected than others. Thus, there is still a need for oral health education to the Kenyan communities to increase the understanding of the possible long-term effects of the IOM practice.

## Abbreviations

CI: Confidence interval
CPQ: Child Perception Questionnaire
Fig.: Figure
HO: Horizontal Overjet
IOM: Infant Oral Mutilation
no.: number
OHRQoL: Oral Health-Related Quality of Life
p: *p*-value used in the statistical testing
SD: Standard Deviation
VO: Vertical Overbite
vs.: versus
yrs.: years

## References

[1] Pindborg JJ (1969). Dental mutilation and associated abnormalities in Uganda. Am J Phys Anthropol.

[2] Hassanali J (2007). Deciduous canine tooth bud removal in infants in East Africa. East Afr Med J.

[3] Girgis S, Gollings J, Longhurst R, Cheng L (2016). Infant oral mutilation – a child protection issue?. Br Dent J.

[4] Vukovic A, Bajsman A, Zukic S, Secic S (2009). Cosmetic dentistry in ancient time – a short review. Bull. Int. Assoc. Paleodontology.

[5] González EL, Pérez BP, Sánchez JAS, Acinas MM (2010). Dental aesthetics as an expression of culture and ritual. Br Dent J.

[6] Babe SPS (1989). The mythology of the killer deciduous canine tooth in southern Sudan. The Journal of Pedodontics.

[7] Garve R, Garve M, Link K, Türp JC, Meyer CG (2016). Infant oral mutilation in East Africa . Therapeutic and ritual grounds. Trop Med Int Health.

[8] Holan G, Mamber E (1994). Extraction of primary canine tooth buds: prevalence and associated dental abnormalities in a group of Ethiopian Jewish children. Int J Paediatr Dent.

[9] Bataringaya A, Ferguson M, Lallo R (2005). The impact of Ebinyo, a form of dental mutilation, on the malocclusion status in Uganda. Community Dent Health.

[10] Hassanali J, Odhiambo JW (2000). Analysis of dental casts of 6-8 and 12-year-old Kenyan children. Eur J Orthod.

[11] Mosha HJ (1983). Dental mutilation and associated abnormalities in Tanzania. Odontostomatolgie Tropicale.

[12] Matee MIN, Van Palerstein Helderman WH. Extraction of ´nylon’teeth and associated abnormalities in Tanzanian children. African Dental Journal 1991;5:21–25. 1819291

[13] Jones A (1992). Tooth mutilation in Angola. Br Dent J.

[14] Welbury RR, Nunn J, Gordon PH, Green-Abate C (1993). “Killer” canine removal and its sequelae in Addis Ababa. Quintessence Int.

[15] Hassanali J, Amwayi P, Muriithi A (1995). Removal of deciduous canine tooth buds in Kenya rural, Maasai Mara. East Afr Med J.

[16] Rodd HD, Davidson LE (2000). ‘Ilko dacowo:’ canine enucleation and dental sequela in Somali children. Int J Paediatr Dent.

[17] Iriso R (2000). ´Killer’canines: the morbidity and mortality of Ebino in northern Uganda. Trop Med Int Health.

[18] Accorsi S, Fabriani M, Ferrarese N, Iriso R, Lukwiya M, Declich S (2003). The burden of traditional practices, Ebino and tea-tea, on child health in northern Uganda. Soc Sci Med.

[19] Kemoli AM (2015). Raising the awareness of infant oral mutilation - myths and facts. Contemporary Clinical Dentistry.

[20] Hassanali J, Amwayi P (1993). Biometric analysis of the dental casts of Maasai following traditional extraction of mandibular permanent central incisors and of Kikyu children. Eur J Orthod.

[21] Khonsari RH, Corre P, Perrin JP, Piot B (2009). Orthodontic consequences of ritual dental mutilations in northern Tchad. J. Oral Maxillofac. Surg..

[22] Connor P. At least a million Sub-Saharan Africans moved to Europe since 2010. 2018. www.PewResearchCenter.org.

[23] Jokovic A, Locker D, Stephens M, Kenny D, Tompson B, Guatt G (2002). Validity and reliability of a questionnaire for measuring child oral-health-related quality of life. J Dent Res.

[24] Foster Page LA, Thomson WM, Jokovic A, Locker D (2005). Validation of the child perceptions questionnaire (CPQ 11-14). J Dent Res.

[25] Bjoerk A (1964). A method for epidemiological registration of malocclusion. Acta Odontologica Scandinavia.

[26] Slade GD, Nuttall N, Sanders AE, Steele JG, Allen PF, Lahti S (2005). Impacts of oral disorders in the United Kingdom and Australia. Br Dent J.

[27] Polder BJ, Van’t hof MA, Van der Linden FP, Kuijpers-Jagtman AM (2004). A meta-analysis of the prevalence of dental agenesis of permanent teeth. Community Dent Oral Epidemiol.

[28] Glendor U (2008). Epidemiology of traumatic dental injuries – a 12 year review of the literature. Dent Traumatol.

[29] Batstone EB, Freer TJ, McNamara JR (2000). Epidemiology of dental trauma: a review of the literature. Aust Dent J.

[30] Rasmussen P, Elhassan E, Raadal M (1992). Enamel defects in primary canines related to traditional treatment of teething problems in Sudan. Int J Paediatr Dent.

[31] Walvekar SV, Qureshi BA (1982). Endemic fluorosis and partial defluoridation of water supplies – a public health concern in Kenya. Community Dent Oral Epidemiol.

[32] Thylstrup A (1983). Posteruptive development of isolated and confluent pits in fluorosed enamel in a 6-year-old girl. Scand J Dent Res.

[33] Helms S (1970). Prevalence of malocclusion in relation to development of the dentition. An epidemiological study of Danish school children. Acta Odontologica Scandinavia.

[34] Helms S (1968). Malocclusion in Danish children with adolescent dentition: an epidemiological study. Am J Orthod.

